# *De novo* assembly of the *Carcinus maenas* transcriptome and characterization of innate immune system pathways

**DOI:** 10.1186/s12864-015-1667-1

**Published:** 2015-06-16

**Authors:** Bas Verbruggen, Lisa K. Bickley, Eduarda M. Santos, Charles R. Tyler, Grant D. Stentiford, Kelly S. Bateman, Ronny van Aerle

**Affiliations:** Biosciences, College of Life & Environmental Sciences, University of Exeter, Geoffrey Pope Building, Exeter, EX4 4QD UK; European Union Reference Laboratory for Crustacean Diseases, Centre for Environment, Fisheries and Aquaculture Science (Cefas), Weymouth Laboratory, Weymouth, Dorset DT4 8UB UK; Aquatic Health and Hygiene Division, Centre for Environment, Fisheries and Aquaculture Science (Cefas), Weymouth Laboratory, Weymouth, Dorset DT4 8UB UK

## Abstract

**Background:**

The European shore crab, *Carcinus maenas*, is used widely in biomonitoring, ecotoxicology and for studies into host-pathogen interactions. It is also an important invasive species in numerous global locations. However, the genomic resources for this organism are still sparse, limiting research progress in these fields. To address this resource shortfall we produced a *C. maenas* transcriptome, enabled by the progress in next-generation sequencing technologies, and applied this to assemble information on the innate immune system in this species.

**Results:**

We isolated and pooled RNA for twelve different tissues and organs from *C. maenas* individuals and sequenced the RNA using next generation sequencing on an Illumina HiSeq 2500 platform. After *de novo* assembly a transcriptome was generated encompassing 212,427 transcripts (153,699 loci). The transcripts were filtered, annotated and characterised using a variety of tools (including BLAST, MEGAN and RSEM) and databases (including NCBI, Gene Ontology and KEGG). There were differential patterns of expression for between 1,223 and 2,741 transcripts across tissues and organs with over-represented Gene Ontology terms relating to their specific function. Based on sequence homology to immune system components in other organisms, we show both the presence of transcripts for a series of known pathogen recognition receptors and response proteins that form part of the innate immune system, and transcripts representing the RNAi, Toll-like receptor signalling, IMD and JAK/STAT pathways.

**Conclusions:**

We have produced an assembled transcriptome for *C. maenas* that provides a significant molecular resource for wide ranging studies in this species. Analysis of the transcriptome has revealed the presence of a series of known targets and functional pathways that form part of their innate immune system and illustrate tissue specific differences in their expression patterns.

**Electronic supplementary material:**

The online version of this article (doi:10.1186/s12864-015-1667-1) contains supplementary material, which is available to authorized users.

## Background

In recent years, large scale sequencing studies have benefitted from the advance of high-throughput sequencing technologies that have resulted in substantial improvement in sequencing efficiency. Additionally, increases in the length and quality of sequencing reads have improved assemblies of sequenced genomes and transcriptomes. Sequencing is a powerful technique allowing for the rapid generation of transcriptome assemblies for any species of interest. Transcriptome sequencing measures expressed sequences only, thus does not have some of the challenges in DNA sequencing (e.g. long repeating sequences) [1]. *De novo* transcriptome assembly removes the need for a reference genome in quantitative RNA-Seq experiments, allowing for the rapid and accurate quantification of transcript abundance in a given biological sample. These aspects are especially useful in studies for organisms with limited genomic resources. Exemplary is the application of *de novo* transcriptome sequencing to a large range of organisms: vertebrates, e.g. brown trout (*Salmo trutta*) [2], invertebrates e.g. sea louse (*Caligus rogercresseyi*) [3], oriental fruit flies (*Bactrocera dorsalis*) [4] and the pollen beetles (*Meligethes aeneus*) [5], fungi (*Trichoderma brevicompactum*) [6] and other microorganisms.

Despite the rapid advances in sequence capabilities and in bioinformatics resources for generating high quality assemblies [7–9], *de novo* transcriptome studies in poorly characterized taxonomic groups continue to be challenging because of difficulties with annotation. This is due to the lack of information available on the genes of interest in closely related organisms. The subphylum Crustacea represents one such taxonomic group for which limited information exists. The Ensembl genome database for metazoan species contains mainly Diptera (flies), Nematoda (worms) and Hymenoptera (ants), but information on only a single crustacean: the common water flea, *Daphnia Pulex* [10]. Furthermore, the number of NCBI Entrez records in the invertebrate taxonomic branch shows huge under-representation of crustaceans. In total, there are approximately 2,300,000 nucleotide sequences in the subphylum Crustacea; in comparison the order Hymenoptera which alone contains almost 2,600,000 nucleotide sequences (numbers dated to April 2014). Consequently, subtaxa within the subphylum Crustacea contain less information: Decapoda (shrimps, crabs, lobsters and crayfish) have a total of 478,358 nucleotide and 44,210 protein sequences available.

The European shore crab (or green crab), *C. maenas,* is a keystone species in the European marine environment and is the only crustacean on the Global Invasive Species Database [11], with invasions into Australia, South Africa and the United States [12]. In such locations, *C. maenas* threatens local fishing industries, for example the destruction of the soft-shell clam (*Mya arenaria*) fishery in New England [13]. *C. maenas* is also an important study species for biomonitoring and ecotoxicology [14, 15]. The species has been used in monitoring for heavy metal contamination [16], metal toxicity studies [17–22], and more recently in exposures studies with nanomaterials [23] and microplastics [24]. Pathological studies are a new area wherein *C. maenas* could play a role. A study investigating infection of crustaceans with White Spot Syndrome Virus (WSSV), recognized as the most significant pathogen affecting global shrimp aquaculture, showed that *C. maenas* are relatively resistant to the virus [25–27]. Despite its importance in these research areas, and its biological significance in the environment, the available molecular resources for *C. maenas* are extremely limited. To date, sequence data for this species comprises approximately 15,000 EST sequences and several hundred nucleotide and protein sequences [28].

Given the ecological importance of *C. maenas,* together with its wider general utility for research purposes, we aimed to sequence, assemble and annotate a shore crab transcriptome. We further set out to establish the relative expression profiles of all sequenced transcripts in different body tissues and organs, and to characterize immune pathways against those known for other invertebrates as a resource for future investigations on the response of this host to pathogens.

## Results and discussion

### RNA sequencing and assembly

Twelve sequence libraries corresponding to 12 pooled tissue samples from adult male and female *C. maenas* were sequenced on an Illumina HiSeq 2500 platform and yielded a total of 138,863,679 paired reads across all tissues. After removal of low quality reads through quality filtering, there were 96,247,762 remaining paired reads. On average 8.0 ± 1.7 million read pairs were obtained for each tissue and the distribution of the reads per pooled transcript sample is presented in Table 1**.** The filtered RNA-Seq data were used for *de novo* transcriptome assembly using the Trinity pipeline with default parameters. The assembled transcriptome encompassed 196,966,469 bp distributed over 153,669 loci, represented by 212,427 transcripts (Table 2). The transcript lengths had a median of 380 bp and a mean of 992 bp (standard deviation = 1363 bp), and ranged between 201 bp and 24,848 bp (Additional file 1 shows the length distribution of assembled transcripts). The transcriptome N50 was calculated to be 2,102 bp. 75.2 % of the read pairs could be mapped back to the *de novo* assembled transcriptome using the bowtie2 aligner.

**Table 1:** Number of read pairs obtained for each crab tissue before and after removal of adapter sequences and quality filtering

Tissue sample	Number of read pairs	Number of clean read pairs
Eggs	9,337,648	6,614,044
Epidermis	11,929,821	8,302,718
Eye	13,463,765	9,430,381
Gill	10,110,102	7,234,304
Haemolymph	10,611,241	7,233,253
Heart	9,657,081	6,717,788
Hepatopancreas	9,216,408	6,471,110
Intestine	8,685,232	5,765,077
Muscle	17,251,355	11,749,555
Nerve	14,278,257	9,670,912
Ovary	11,125,170	7,869,190
Testis	13,197,599	9,189,430
Total	138,863,679	96,247,762

**Table 2:** Transcriptome statistics

Description	Value
Number of loci	153,669
Number of transcripts	212,427
Maximum transcript length (bp)	24,848
Minimal transcript length (bp)	201
Mean transcript length (bp)	992
Standard deviation (bp)	1363
Median transcript length (bp)	380
Total length (bp)	196,966,469
N50 (bp)	2,102

A total of 231 out of the 248 highly conserved eukaryotic “core” genes were identified completely (93.15 %) and 245 genes (98.79 %) partially in the transcriptome by the CEGMA pipeline [29], indicating that the transcriptome contains a near complete set of core eukaryotic genes.

### Transcriptome characterization

Several approaches were taken to annotate the assembled transcripts. Firstly, the transcript sequences were compared to existing *C. maenas* EST sequences in the NCBI database using BLASTn. In total, 19,981 sequences (9.4 % of the total number of transcripts) showed high similarity to 4,759 EST sequences (30.6 % of total *C. maenas* ESTs in NCBI; Table 3). This indicates that the majority of transcripts in the assembly were previously un-reported for *C. maenas*. A broader sequence homology search was performed using BLASTx against the NCBI non-redundant *nr* protein database and hits were found for 62,804 (29.6 %) of the transcripts using an e-value threshold of 1e-3. Open reading frames were identified in 58,383 (27.5 %) of transcripts and the majority of the predicted peptides (41,108), corresponding to 70.4 % of all predicted peptides were annotated using the UniProt/Swissprot database (with an e-value cut-off of 1e-5). Furthermore, conserved Pfam domains were assigned to 37,776 (67.4 %) of the peptides and 4,132 (1.9 %) of these peptides appeared to contain signal peptides (Table 3) as determined by SignalP. Transcriptome annotation details can be found in Additional files 2 and 3.

**Table 3:** Number of annotated transcripts and open reading frames (identified by TransDecoder) using different annotation methods and sequence databases

Input	Annotation method	Number of annotated transcripts
All transcripts	BLASTx – NCBI nr protein	62,804 (29.6 %)
All transcripts	BLASTn – *C.maenas* EST	19,891 (9.4 %)
All transcripts	BLAST2GO	8,091 (3.8 %)
All transcripts	TransDecoder ORF finder	58,383 (27.5 %)
All transcripts	KEGG	30,352 (14.3 %)
Open reading frames	BLASTp – UniProt/SwissProt	41,108 (70.4 %)
Open reading frames	Pfam	37,776 (67.4 %)
Open reading frames	SignalP	4,132 (1.9 %)
Open reading frames	TmHMM	0 (0.0 %)

### Transcriptome functional annotation

Gene Ontology (GO) terms were assigned to 53,766 (25.3 %) of the annotated transcripts and 47.23 % of the annotated predicted peptides (UniProt/Swissprot; Table 3) by BLAST2GO [30]. The most common GO terms were protein binding (10.93 %), cytoplasm (10.93 %), nucleus (10.07 %), plasma membrane (6.55 %) and membrane (6.25 %). The most common annotations for the three gene ontology trees are presented in Table 4, and a full list of transcript annotations is available in Additional file 4.

**Table 4:** Identification, sequence similarity and Gene Ontology annotation statistics of peptide sequences in the transcriptome

Description		Number of sequences	Percentage of sequences (%)
Transcripts		212,427	
TransDecoder peptides		58,383	
Peptides with Swissprot/Uniprot annotation		41,108	70.41
GO annotated transcripts		53,766	25.31
GO annotated peptides		19,423	47.23
GO tree	GO	Count	%
Cellular Component	cytoplasm	2,122	10.9
	nucleus	1,955	10.1
	plasma membrane	1,272	6.6
	membrane	1,213	6.3
	cytosol	1,195	6.2
Molecular Function	protein binding	2,577	13.3
	binding	1,071	5.5
	ATP binding	755	3.9
	metal ion binding	569	2.9
	protein homodimerization activity	485	2.5
Biological Process	cellular process	597	3.1
	regulation of cellular process	539	2.8
	primary metabolic process	446	2.3
	response to stimulus	419	2.2
	transport	416	2.1

### Taxonomy

**Fig. 1:**
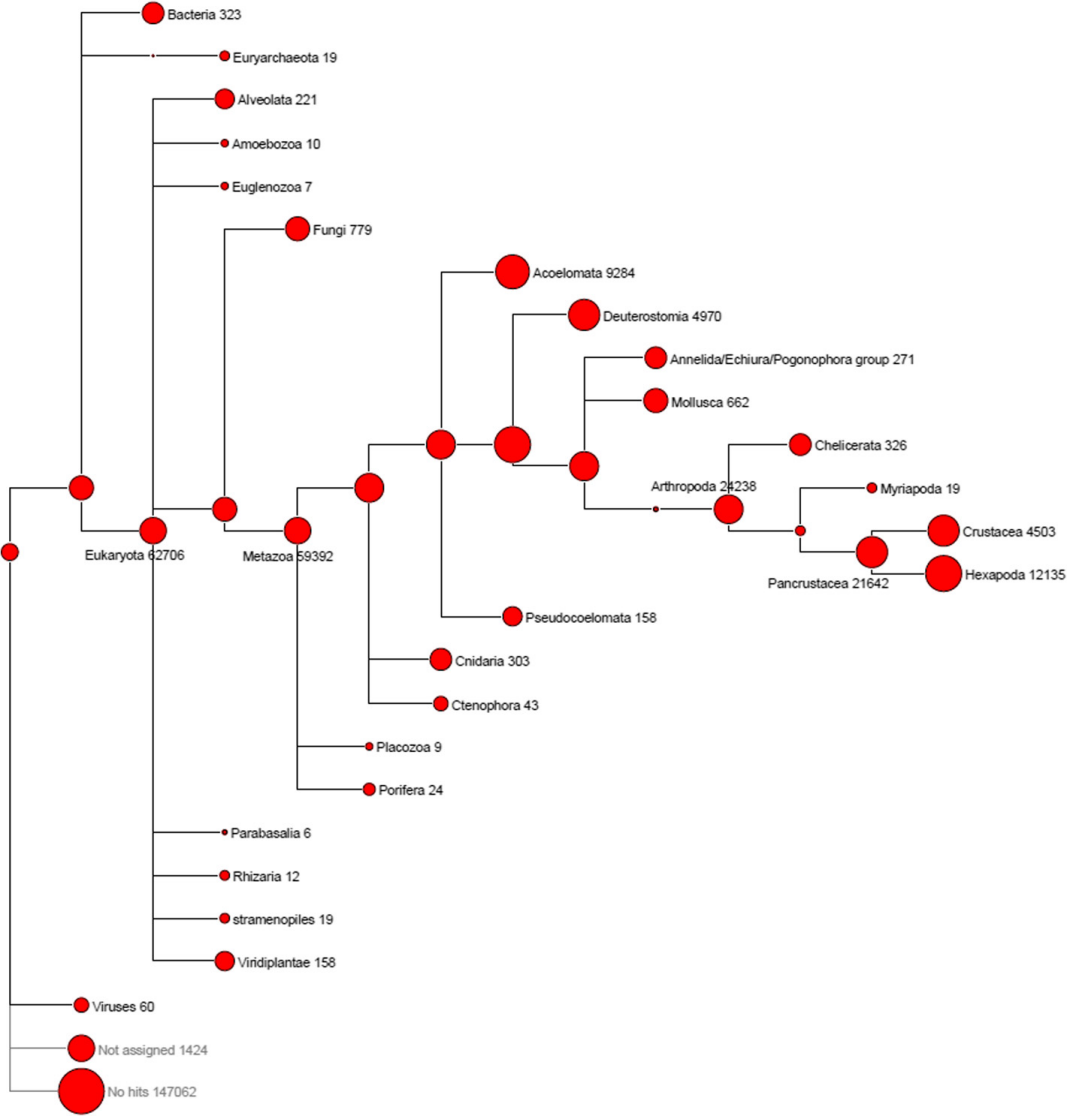
Taxonomic classifications of *Carcinus maenas* transcripts. Partially collapsed phylogenetic tree produced by MEGAN4. Numbers illustrate the number of transcripts representing each taxa. Within the metazoan taxon, the pancrustacea represented the largest taxonomic group

The BLASTx output was used as input for MEGAN4 to illustrate the taxonomic origin of BLAST hits for the transcriptome in a phylogenetic tree. A partially collapsed phylogenetic tree is presented in Fig. 1. The taxon with the largest number of sequence homologies was the pancrustacean taxon wherein 21,642 *C. maenas* transcripts showed similarity. Within this taxon, transcripts were split between the crustacean and hexapoda taxa. Since *C. maenas* is a crustacean species it is expected that a large proportion of transcripts show similarity to sequences derived from this taxon. However, due to the limitations in crustacean genomic resources a significant proportion of transcripts mapped to related sequences in the hexapoda taxon instead (containing e.g. *Drosophila melanogaster*). Furthermore, it can be seen that a variety of sequences were derived from micro-organisms (e.g. bacteria, fungi and viruses), which may correspond to transcripts originating from micro-organisms living within the *C. maenas* hosts, and/or may reflect contamination of kits and samples with environmental micro-organisms [31]. To remove these potential contaminating transcripts from the transcriptome we filtered the transcriptome for sequences that mapped to the metazoan taxon. Following the application of this filtering step, a transcriptome encompassing 59,392 transcripts was retained and used in subsequent analysis.

### Differential gene expression

Transcript expression in the twelve tissue types was estimated by the RSEM program [32]. Next, differentially expressed transcripts were identified through comparing gene expression profiles of each sampled tissue to the others. The number of differentially expressed (metazoan) transcripts for the various tissues ranged between 1,223 in gill and 2,741 in hepatopancreas (FDR < 0.01; Table 5). All tissues showed enrichment for Gene Ontology (GO) terms; the top five for every tissue are listed in Table 6 (a complete list is presented in Additional file 5). The enriched GO terms often reflected the function of the tissue e.g. structural constituent of cuticle in eggs, angiogenesis in haemolymph and sarcolemma in muscle. In several tissues the link to function is not very clear in the top five, but becomes apparent in other enriched terms. For example, in the eye, phototransduction (FDR = 9.42e−4) and detection of light stimulus (FDR = 1.01e−3) were over-represented; contractile fibre (FDR = 6.72e−3) and sarcomere (FDR = 7.15e−3) were enriched in the heart tissue and finally, the epidermis and ovary tissues yielded only three enriched annotations (Table 6).

**Table 5:** Differentially expressed transcripts in specific tissues

Tissue	Differentially expressed transcripts
eggs	1,605
epidermis	1,339
eye	1,312
gill	1,223
Haemolymph	2,008
heart	1,226
hepatopancreas	2,741
intestine	1,519
muscle	2,200
nerve	1,989
ovary	1,751
testis	1,391

**Table 6:** Top 5 most enriched Gene Ontology terms in specific tissues

Tissue	GO-ID	Term	*P* - value	FDR
Eggs	GO:0042302	structural constituent of cuticle	9.86e-11	1.07e-6
Eggs	GO:0003677	DNA binding	5.25e-7	2.86e-3
Eggs	GO:0006260	DNA replication	2.78e-6	1.01e-2
Eggs	GO:0006261	DNA-dependent DNA replication	5.76e-6	1.57e-2
Eggs	GO:0001708	cell fate specification	1.06e-5	2.30e-2
Epidermis	GO:0018298	protein-chromophore linkage	3.85e-7	2.56e-3
Epidermis	GO:0015772	oligosaccharide transport	7.05e-7	2.56e-3
Epidermis	GO:0015766	disaccharide transport	7.05e-7	2.56e-3
Eye	GO:0003008	system process	7.00e-12	7.61e-8
Eye	GO:0050877	neurological system process	2.36e-11	1.28e-7
Eye	GO:0022834	ligand-gated channel activity	9.64e-9	2.62e-5
Eye	GO:0015276	ligand-gated ion channel activity	9.64e-9	2.62e-5
Eye	GO:0070011	peptidase activity, acting on L-amino acid peptides	1.57e-8	3.42e-5
Gill	GO:0070160	occluding junction	1.71e-6	4.20e-3
Gill	GO:0005344	oxygen transporter activity	1.84e-6	4.20e-3
Gill	GO:0015671	oxygen transport	1.84e-6	4.20e-3
Gill	GO:0015669	gas transport	1.84e-6	4.20e-3
Gill	GO:0005923	tight junction	2.65e-6	4.20e-3
Haemolymph	GO:0001525	angiogenesis	5.76e-11	6.27e-7
Haemolymph	GO:0048514	blood vessel morphogenesis	1.80e-9	9.79e-6
Haemolymph	GO:0001568	blood vessel development	1.25e-8	4.54e-5
Haemolymph	GO:0001944	vasculature development	3.97e-8	1.08e-4
Haemolymph	GO:0009653	anatomical structure morphogenesis	1.46e-7	1.65e-4
Heart	GO:0016328	lateral plasma membrane	1.78e-7	1.94e-3
Heart	GO:0006768	biotin metabolic process	1.28e-6	3.04e-3
Heart	GO:0004736	pyruvate carboxylase activity	1.28e-6	3.04e-3
Heart	GO:0005344	oxygen transporter activity	1.67e-6	3.04e-3
Heart	GO:0015671	oxygen transport	1.67e-6	3.04e-3
Hepatopancreas	GO:0016491	oxidoreductase activity	6.35e-16	6.91e-12
Hepatopancreas	GO:0003824	catalytic activity	2.87e-11	1.56e-7
Hepatopancreas	GO:0044710	single-organism metabolic process	2.06e-10	7.47e-7
Hepatopancreas	GO:0005576	extracellular region	5.68e-10	1.20e-6
Hepatopancreas	GO:0005764	lysosome	6.61e-10	1.20e-6
Intestine	GO:0016337	cell-cell adhesion	5.72e-9	6.22e-5
Intestine	GO:0005548	phospholipid transporter activity	7.29e-8	3.97e-4
Intestine	GO:0006022	aminoglycan metabolic process	1.56e-7	4.06e-4
Intestine	GO:0015917	aminophospholipid transport	2.09e-7	4.06e-4
Intestine	GO:0004012	phospholipid-translocating ATPase activity	2.09e-7	4.06e-4
Muscle	GO:0042383	sarcolemma	1.93e-11	2.10e-7
Muscle	GO:0031674	I band	7.82e-11	4.25e-7
Muscle	GO:0006811	ion transport	2.87e-10	1.04e-6
Muscle	GO:0030018	Z disc	1.94e-9	5.29e-6
Muscle	GO:0044449	contractile fiber part	2.54e-9	5.52e-6
Nerve	GO:0015277	kainate selective glutamate receptor activity	1.39e-14	1.51e-10
Nerve	GO:0004872	receptor activity	2.54e-12	1.38e-8
Nerve	GO:0048172	regulation of short-term neuronal synaptic plasticity	5.16e-12	1.87e-8
Nerve	GO:0004970	ionotropic glutamate receptor activity	1.02e-11	2.77e-8
Nerve	GO:0048168	regulation of neuronal synaptic plasticity	4.92e-11	1.07e-7
Ovary	GO:0016459	myosin complex	1.43e-7	1.56e-3
Ovary	GO:0018298	protein-chromophore linkage	1.67e-6	9.10e-3
Ovary	GO:0036002	pre-mRNA binding	1.37e-5	4.96e-2
Testis	GO:0008499	UDP-galactose:beta-N-acetylglucosamine beta-1,3-galactosyltransferase activity	5.07e-17	5.52e-13
Testis	GO:0035250	UDP-galactosyltransferase activity	1.45e-16	7.86e-13
Testis	GO:0005797	Golgi medial cisterna	1.38e-15	5.00e-12
Testis	GO:0048531	beta-1,3-galactosyltransferase activity	6.12e-15	1.66e-11
Testis	GO:0008378	galactosyltransferase activity	1.26e-14	2.75e-11

### Immune pathway characterization in *C. maenas*

Application of *C. maenas* as a model organism to study crustacean infectious diseases requires insight in the organism's immune system. Since crustaceans do not have adaptive immune systems, innate immune strategies will predominate in this organism when responding to pathogenic insults. We investigated the presence of several innate immune system pathways in the *C. maenas* transcriptome and mapping the transcripts to pathways in the KEGG database. In total 30,352 (14.3 %) of transcripts were annotated to a KEGG orthology group (Table 3). The KEGG server [33] allows mapping of the present orthology groups to pathways in the KEGG database and visualization of presence/absence of their components. Li *et al.* 2013 characterized a selection of innate immune pathways in the hepatopancreas transcriptome of the mitten crab *Eriocheir sinensis*, including the RNAi pathway, Toll-like receptor pathway, immune deficiency (IMD) pathway, the JAK-STAT and mitogen activated protein kinase (MAPK) signalling pathways [34]. We characterized the same pathways in the *C. maenas* transcriptome with additions including the endocytosis pathway. The latter is not directly related to the immune response but many viruses utilize its machinery to gain entry to host cells [35]. Its characterization can thus be important for investigations of viral infections.

### Pathogen associated molecular pattern recognition

The first stage in immune defence is the identification of invading pathogens by an organism. In this process a distinction between cells from the organism itself and those of the invading pathogens needs to occur. To achieve this, the innate immune system employs a group of pattern recognition receptors (PRRs) that are able to recognize pathogen associated molecular patterns (PAMPs). Examples of PAMPs include lipopolysaccharides, peptidoglycans and β-1,3-glucans [36] and groups of PRRs include gram-negative binding proteins (GNBPs), peptidoglycan recognition proteins (PGRP), thioester containing proteins and lectins [36]. Upon successful pathogen recognition, PRRs initiate immune responses.

*C. maenas* transcripts that show sequence similarity to known PRR groups are shown in Table 7. Representatives of most groups of PRR have counterparts in the *C. maenas* transcriptome as identified through sequence similarity, often to sequences derived from organisms that are closely related to *C. maenas*. One group that is not represented are the PGRPs, this has also been reported in other crustacean species [37, 38]. Down syndrome cell adhesion molecule (Dscam) is a PAMP recognition protein that has been hypothesized to be involved in immune memory (reviewed in Armitage et al. 2014 [39]). This gene can produce many isoforms, and initial findings suggested that it played an important role in the development of the nervous system in invertebrates where Dscam isoforms aid in the discrimination of neuritis [39]. Dscam isoforms were later found to be able to recognise pathogens, aiding in phagocytosis [40]. In concordance with this hypothesis, the *C. maenas* Dscam gene appears to encode many isoforms, and in total 242 transcripts with significant similarity to Dscam sequences in NCBI were found in the transcriptome.

**Table 7:** *Carcinus maenas* pathogen associated molecular pattern recognition genes

PRP group	Transcript	Identity (%)	Length	E-value	Query	Ancestor
GNBP	comp44152_c0_seq1	42.06	340	1.00e-65	gi|300507044 : gram-negative binding protein [*Artemia sinica*]	Crustacea
	comp44453_c0_seq (1–2)	58.06	341	3.00e-123	gi|62122584 : GNBP [*Oryzias latipes*]	Bilateria
	comp74133_c0_seq1	44.8	346	8.00e-88	gi|62122584 : GNBP [*Oryzias latipes*]	Bilateria
	comp83740_c0_seq (1–5)	46.02	339	5.00e-87	gi|62122584 : GNBP [*Oryzias latipes*]	Bilateria
	comp19734_c0_seq1	41.55	142	8.00e-32	gi|62122584 : GNBP [*Oryzias latipes*]	Bilateria
	comp136078_c0_seq1	62.96	81	3.00e-26	gi|62122584 : GNBP [*Oryzias latipes*]	Bilateria
	comp75261_c0_seq1	27.57	243	6.00e-22	gi|62122584 : GNBP [*Oryzias latipes*]	Bilateria
TECP	comp85313_c2_seq1	39.94	318	6.00e-63	gi|385049105 : thioester containing protein 3, partial [*Daphnia parvula*]	Crustacea
	comp65627_c0_seq1	46.34	246	3.00e-58	gi|54644242 : Thioester-containing protein 6 [*Drosophila pseudoobscura pseudoobscura*]	Pancrustacea
	comp87629_c0_seq4	74.36	234	6.00e-101	gi|331031264 : TEP isoform 2 [*Pacifastacus leniusculus*]	Pleocyemata
	comp74624_c1_seq1	40.65	310	8.00e-56	gi|385049099 : thioester containing protein 3, partial [*Daphnia pulex*]	Crustacea
	comp65627_c1_seq1	36.78	590	6.00e-118	gi|54644242 : Thioester-containing protein 6 [*Drosophila pseudoobscura pseudoobscura*]	Pancrustacea
	comp74624_c2_seq1	37.83	534	1.00e-105	gi|54644242 : Thioester-containing protein 6 [*Drosophila pseudoobscura pseudoobscura*]	Pancrustacea
	comp85313_c0_seq1	38.14	430	1.00e-80	gi|568250870 : thioester-containing protein [*Anopheles darlingi*]	Pancrustacea
	comp103781_c0_seq1	43.36	113	4.00e-22	gi|54644242 : Thioester-containing protein 6 [*Drosophila pseudoobscura pseudoobscura*]	Pancrustacea
C-Type Lectin	comp69837_c0_seq1	43.15	146	1.00e-25	gi|558633447 : C-type lectin [*Marsupenaeus japonicus*]	Decapoda
	comp86095_c0_seq (1–2)	43.92	148	2.00e-25	gi|558633447 : C-type lectin [*Marsupenaeus japonicus*]	Decapoda
	comp68699_c0_seq1	38.89	144	1.00e-24	gi|558633447 : C-type lectin [*Marsupenaeus japonicus*]	Decapoda
	comp87731_c3_seq (2–3)	33.78	225	8.00e-25	gi|657397985 : C-type lectin receptor-like tyrosine-kinase plant [*Medicago truncatula*]	Eukaryota
	comp88573_c0_seq (1–2)	57.5	80	4.00e-22	gi|676264911 : C-type lectin domain family 3 member A [*Fukomys damarensis*]	Bilateria
	comp90611_c0_seq1	64.56	158	5.00e-60	gi|575878533 : C-type lectin [*Scylla paramamosain*]	Portunoidea

The immune responses initiated by these PRRs can occur at a transcriptional level, e.g. activation of Toll and IMD can aid in phagocytosis e.g. Dscam binding, or can initiate proteolytic cascades leading to melanization.

### Toll-like receptor pathway

The Toll receptor pathway is a signalling route that responds to the presence of PAMPs by ultimately activating Nf-κB [41]. In mammals, Toll-like receptors (TLR) bind to PAMPs resulting in dimerization. Upon forming dimers, the TLRs recruit MyD88 and subsequently IRAK kinases. After IRAK kinases activate TRAF6, its binding to TAK1 and IKKβ ultimately frees Nf-κB to diffuse into the nucleus [42]. In invertebrates, such as *D. melanogaster,* the mechanism is slightly different, and instead of directly binding PAMPs, TLRs respond to the Toll ligand Spätzle [41].

**Fig. 2:**
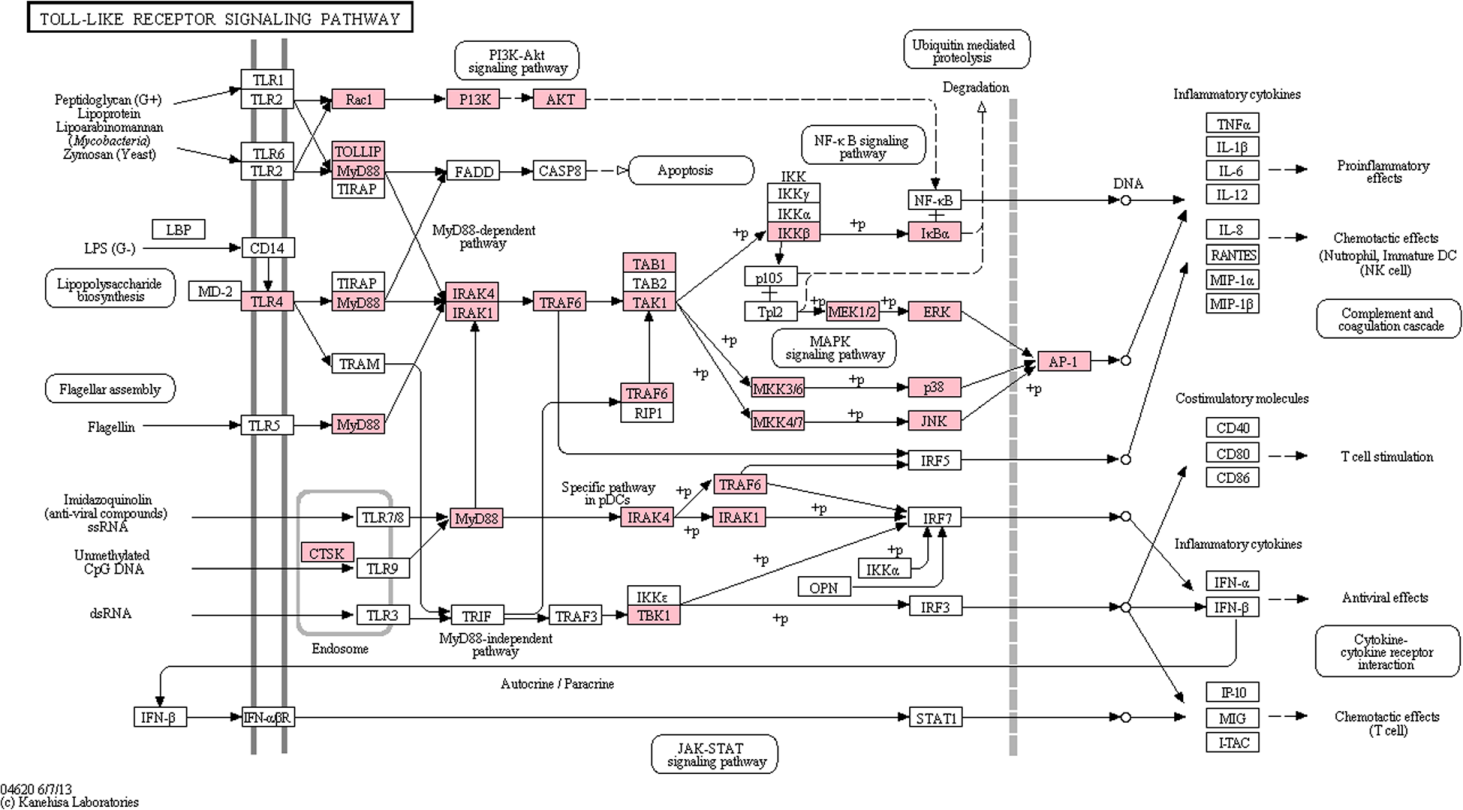
Toll-like receptor signalling pathway coverage. The Toll-like receptor signalling pathway in the KEGG database. Proteins in the pathway are depicted by boxes while arrows depict signalling routes. Pathway components with homologues in the *Carcinus maenas* transcriptome are highlighted in pink

The KEGG database contains a version of the Toll-like receptor pathway which was used to visualize the coverage of this pathway in the *C. maenas* transcriptome (see Fig. 2). Homologues were found for most of the components in the paths from TLR to NF-κB and activator protein-1 (AP-1). Since KEGG is targeted towards vertebrate genes and pathways, a characterization of an invertebrate Toll signalling pathway was also performed (see Methods for pathway analysis strategy). Components of the *D. melanogaster* Toll signalling pathway were taken from Li *et al.* [34] and Kingsolver *et al.* [41] and investigated for presence and expression in the assembled transcriptome. Transcripts with significant sequence similarity to most of the Toll pathway components were found in the transcriptome (Additional file 6). Tube, an IRAK homolog, was not identified in the *C. maenas* transcriptome. Successfully identified transcripts were found to be expressed across all tissues (Additional file 7), and the median expression values varied from 82.4 FPKM for myD88 to 5576.7 FPKM for Toll.

### IMD pathway

**Fig. 3:**
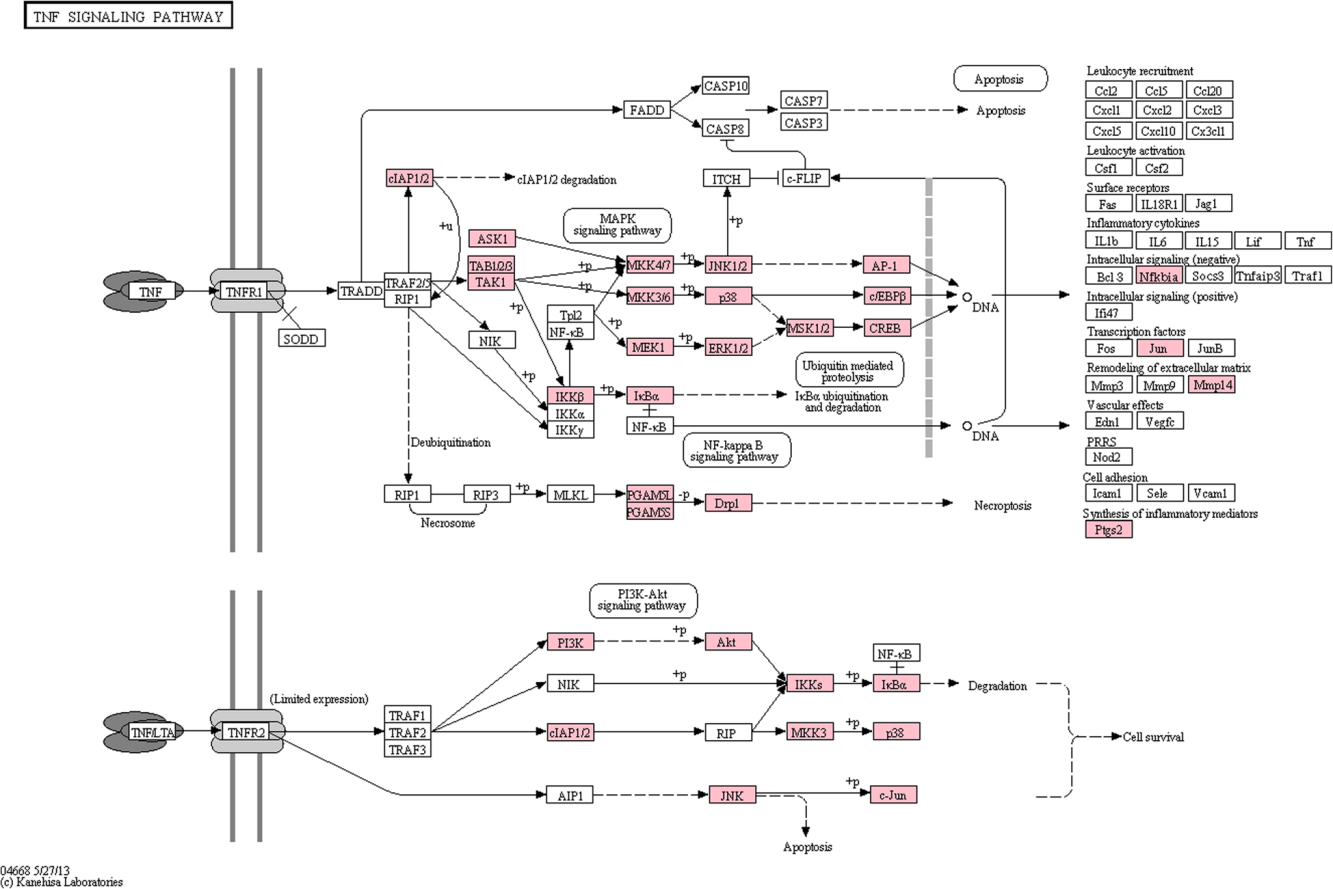
TNF signalling pathway. Overview of the KEGG TNF signalling pathway, components depicted as in Fig. 2. Components with homologues in the *Carcinus maenas* transcriptome are highlighted in pink

The IMD pathway is also activated upon pathogen recognition, in particular by Gram-negative bacteria. Similar to Toll-like receptors, the binding of peptidoglycan by PGRPs leads to dimerization [41]. After the dimerization, the signal is transmitted through IMD, as well as FADD and DREDD. Activation of DREDD leads to poly-ubiquitination of IMD [41], binding of TAK1 and assembly of the IKK complex. Relish phosphorylation is promoted by IKK, and an event followed by cleavage of Relish by DREDD cause translocation of the N-terminal end to the nucleus where it regulates the expression of effector molecules [41]. Since the KEGG database does not contain the IMD pathway, the KEGG TNF-signalling pathway was used instead. As for the Toll-like receptor pathway, homologues also were found for most constituents of the TNF-signalling pathway (see Fig. 3). Manual identification of IMD pathway components derived from Kingsolver *et al.* [41] showed that FADD was the only absent component in the *C. maenas* transcriptome (Additional file 6). IMD itself was only expressed in three out of twelve tissues (eye, ovary and haemolymph) whereas the rest of the IMD pathway was expressed across all tissue types (Additional file 8).

### JAK-STAT signalling pathway

**Fig. 4:**
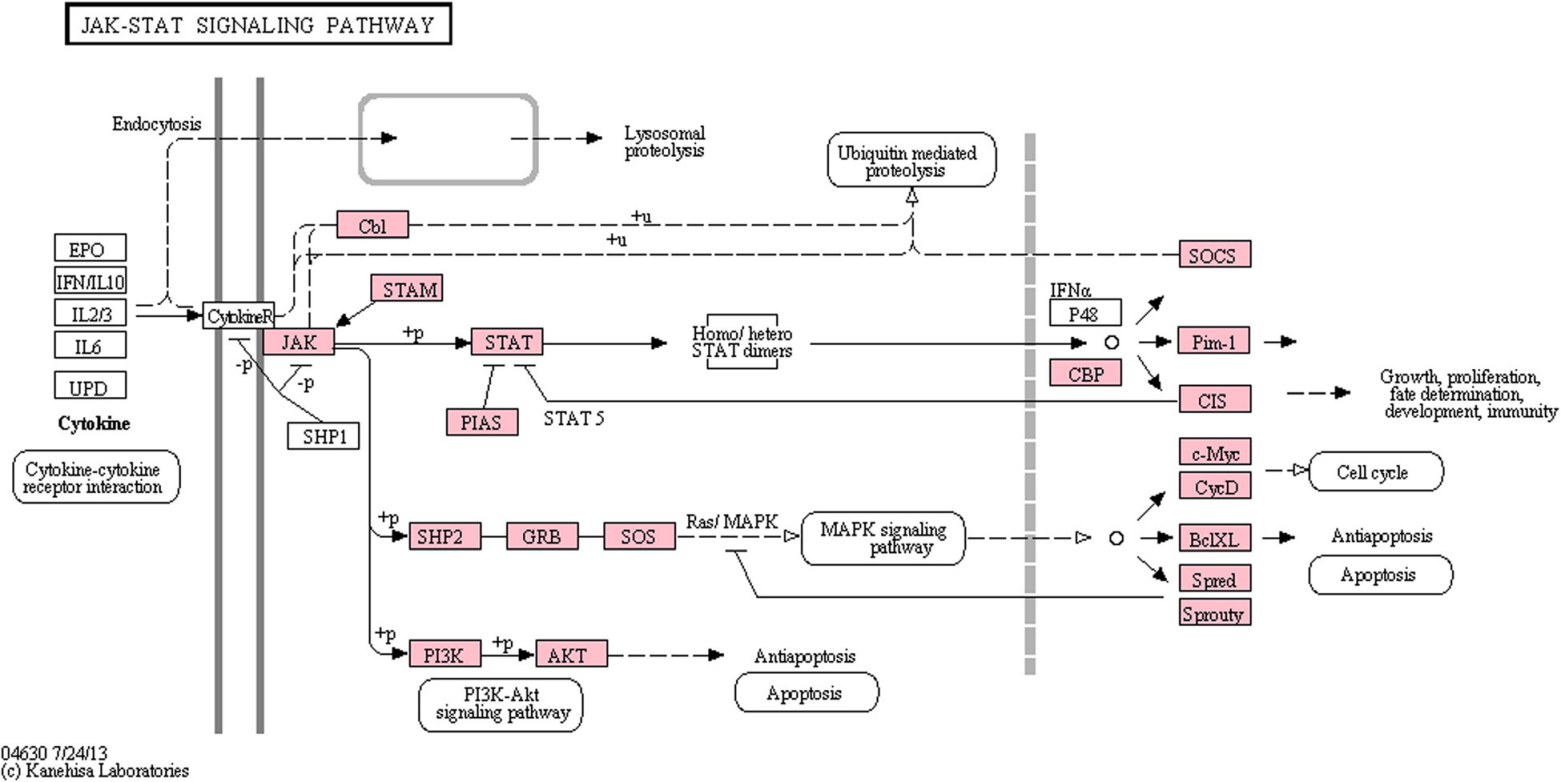
JAK-STAT signalling pathway. Overview of the KEGG JAK-STAT signalling pathway. Components with homologues in the *Carcinus maenas* transcriptome are highlighted in pink

The JAK-STAT signalling pathway mediates the response to chemical messenger molecules like cytokines. It has been shown that STAT signalling is activated upon WSSV infection in shrimp [43]. JAK tyrosine kinases bind to cytokine receptors and upon ligand binding they phosphorylate tyrosine residues on those receptors [44]. STAT is able to bind and subsequently be phosphorylated by JAK [44]. Following phosphorylation, STAT forms dimers, translocates to the nucleus and organizes the response to the signalling molecule by altering gene expression [44]. Inhibitors of JAK-STAT signalling are present at several stages and include dominant negative co-receptors, prevention of STAT recruitment by SOCS (suppressor of cytokine signalling) and protein inhibitors of activated STAT (PIAS) [44]. The KEGG reference pathway and coverage in the transcriptome are presented in Fig. 4. Most of the components of the JAK-STAT pathway have a homologue in the *C. maenas* transcriptome. The pathway in Fig. 4 shows that only the cytokine receptor was not identified by the KEGG annotation. However one transcript (comp79993_c0_seq2) showed highly significant sequence homology to the cytokine receptor of *Harpegnathos saltator* (e = 3.00e−74) and the domeless receptor of *Tribolium castaneum* (e = 2.00e−51).

### Response proteins

The signalling cascade through the IMD, Toll and JAK-STAT pathways results in a transcriptional immune response mediated by transcription factors like STAT and NF-kB. One part of this immune response includes antimicrobial peptides (e.g. anti-lipopolysaccharide factor (ALF) and lysozyme), which have evolved to attack pathogens [45, 36]. In addition to antimicrobial peptides, the innate immune system also employs nitric oxide as a defensive molecule. Nitric oxide is an important redox activated signalling molecule and can be produced in large concentrations by nitric oxide synthase 2 (NOS-2), an enzyme synthesized as a response to PRR activation [46]. Response proteins identified in the *C. maenas* transcriptome are listed in Table 8 along with their target pathogen type, as described in Tassanakajon et al. [45]. Neither penaeidins [47] nor stylicins [48] were identified for *C. maenas* and we hypothesise that both are probably limited to penaeid shrimp species. The antimicrobial arsenal of *C. maenas* includes ALF, lysozyme, crustins, carcinin and inducible nitric oxide synthase. It is possible that the *C. maenas* transcriptome also contains novel anti-microbial peptides but to identify them will require exposure studies to trigger their activation.

**Table 8:** *Carcinus maenas* Immune system response proteins

Response protein	Transcript	Identity (%)	Length	E-value	Query	Ancestor
ALF	comp79835_c0_seq2	65.98	97	2.00e-34	gi|302138013 : anti-lipopolysacharide factor [*Fenneropenaeus indicus*]	Decapoda
Crustin	comp88229_c1_seq1	56.36	110	8.00e-31	gi|162945361 : crustin antimicrobial peptide [*Scylla paramamosain*]	Portunoidea
	comp91133_c0_seq1	65.38	78	7.00e-24	gi|255653868 : crustin 1 [*Panulirus japonicus*]	Pleocyemata
Carcinin	comp88229_c1_seq1	86.36	110	1.00e-49	gi|18157188 : carcinin [*Carcinus maenas*]	Carcinus maenas
Lysozyme	comp83352_c1_seq4	41.13	124	4.00e-23	gi|675374133 : Lysozyme 1, partial [*Stegodyphus mimosarum*]	Arthropoda
	comp83352_c1_seq2	41.13	124	4.00e-23	gi|675374133 : Lysozyme 1, partial [*Stegodyphus mimosarum*]	Arthropoda
	comp83352_c1_seq1	41.13	124	4.00e-23	gi|675374133 : Lysozyme 1, partial [*Stegodyphus mimosarum*]	Arthropoda
iNOS	comp89503_c2_seq (1–26)	52.6	308	1.00e-96	gi|13359094 : nitric oxide synthase 2 [*Meriones unguiculatus*]	Pancrustacea

### Melanization pathway

**Fig. 5:**
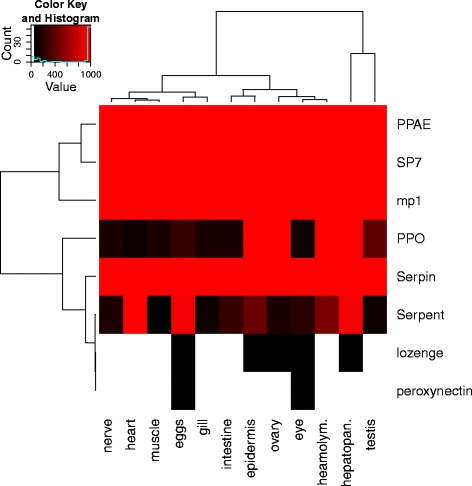
Melanization pathway expression. Expression of melanization pathway components in twelve *Carcinus maenas* tissues. The expression values are presented in FPKM, values of 0 are coloured white and values over 10000 FPKM are binned together

The *C. maenas* innate immune system also contains a more direct response to pathogen infection in the form of the melanization pathway. Activated within minutes after infection, melanization damages and encapsulates invading pathogens with melanin [49]. The production of melanin from phenols and quinones generates reactive oxygen species that are damaging to the pathogen. Synthesis of quinones is catalyzed by the phenol oxidase (PO) enzyme. PO is readily available as a precursor (proPO) that is activated through proteolysis, ensuring a fast response time. Recognition of PAMPs by PRRs leads to activation of a serine protease cascade that ends with the activation of PO [45, 49, 50]. The proteolytic cascade is regulated by serpins that act as serine protease inhibitors [49]. Members of the melanization pathway as described in Tang 2009 [49] and transcripts with significant sequence similarity are listed in Additional file 6. The upstream proteases of proPO: MP1, Sp7 and the activating enzyme PPAE and prophenoloxidase itself are identified. Transcripts coding for the transcription factors serpent and lozenge, controlling the expression of proPO [49], and Peroxinectin, a protein that is associated with the proPO pathway and aids in cellular adhesion of haemocytes to pathogens [51] were also found. The expression of proPO varied across tissues (see Fig. 5), and was particularly high in the hepatopancreas and ovary.

### RNAi pathway

RNA interference (RNAi) is one of the major antiviral pathways within the invertebrate innate immune system [52]. The pathway produces small interfering RNA molecules (siRNAs) from virus derived dsRNA [41]. In short, dsRNA is recognized by Dicer proteins that subsequently cleave it to 21 nucleotide (nt) siRNAs. siRNAs are loaded into the RISC complex, which utilizes argonaute (Ago) protein to cleave viral RNAs targeted by the siRNA, and thus silencing expression [41]. The RNAi pathway can also be employed to silence specific genes in cells and forms the basis of antiviral immunity strategies, a topic explored in La Fauce *et al.* 2012 [53]. Identification of components of the RNAi pathway was based on those listed in Wang *et al.* 2014 [52], results are shown in Table 9. *D. melanogaster* has distinct functions for dicer-1 and dicer-2, the first being involved in the miRNA pathway and the latter in siRNA [54, 41, 55]. Both dicer-1 and dicer-2 were identified in *C. maenas* suggesting that a similar division of tasks could exist in this organism.

**Table 9:** *Carcinus maenas* RNAi pathway components

RNAi	Transcript	Identity (%)	Length	E-value	Query	Ancestor
TRBP	comp79785_c0_seq (1–2)	83.97	343	2.00e-167	gi|332271591 : TAR RNA-binding protein isoform 1 [*Marsupenaeus japonicus*]	Decapoda
	comp79200_c0_seq (1–2)	36.74	460	4.00e-77	gi|110825988 : probable methyltransferase TARBP1 [*Homo sapiens*]	Bilateria
	comp49673_c0_seq1	46.34	205	2.00e-41	gi|444174849 : TAR RNA-binding protein 1 [*Penaeus monodon*]	Decapoda
R2D2	comp79785_c0_seq (1–2)	48.86	350	8.00e-81	gi|619831236 : R2D2 [*Bemisia tabaci*]	Pancrustacea
	comp49673_c0_seq1	38.32	167	6.00e-24	gi|619831236 : R2D2 [*Bemisia tabaci*]	Pancrustacea
drosha	comp87202_c0_seq1	93.37	829	0	gi|396941645 : drosha [*Marsupenaeus japonicus*]	Decapoda
Dicer2	comp90354_c0_seq (1–11)	47.73	1253	0	gi|402534262 : Dicer-2 [*Marsupenaeus japonicus*]	Decapoda
Dicer1	comp85246_c1_seq1	77.95	1578	0	gi|195424855 : dicer-1 [*Litopenaeus vannamei*]	Decapoda
	comp90354_c0_seq (5–6)	31	658	1.00e-83	gi|195424855 : dicer-1 [*Litopenaeus vannamei*]	Decapoda
	comp55144_c0_seq1	61.06	113	3.00e-37	gi|283827860 : dicer-1 [*Marsupenaeus japonicus*]	Decapoda
	comp77864_c(1–2)_seq (1–2)	83.67	98	2.00e-40	gi|195424855 : dicer-1 [*Litopenaeus vannamei*]	Decapoda
ago2	comp81967_c(1–2)_seq1	37.16	802	5.00e-139	gi|563729913 : argonaute2 [*Penaeus monodon*]	Decapoda
	comp41784_c0_seq1	52.74	876	0	gi|563729913 : argonaute2 [*Penaeus monodon*]	Decapoda
	comp76466_c0_seq1	42.51	821	0	gi|563729913 : argonaute2 [*Penaeus monodon*]	Decapoda
ago1	comp81967_c1_seq1	89.45	758	0	gi|321468117 : putative Argonaute protein [*Daphnia pulex*]	Crustacea
	comp41784_c0_seq1	43.65	811	0	gi|321468117 : putative Argonaute protein [*Daphnia pulex*]	Crustacea
	comp76466_c0_seq1	41.58	671	4.00e-148	gi|321468117 : putative Argonaute protein [*Daphnia pulex*]	Crustacea

### Endocytosis pathway

**Fig. 6:**
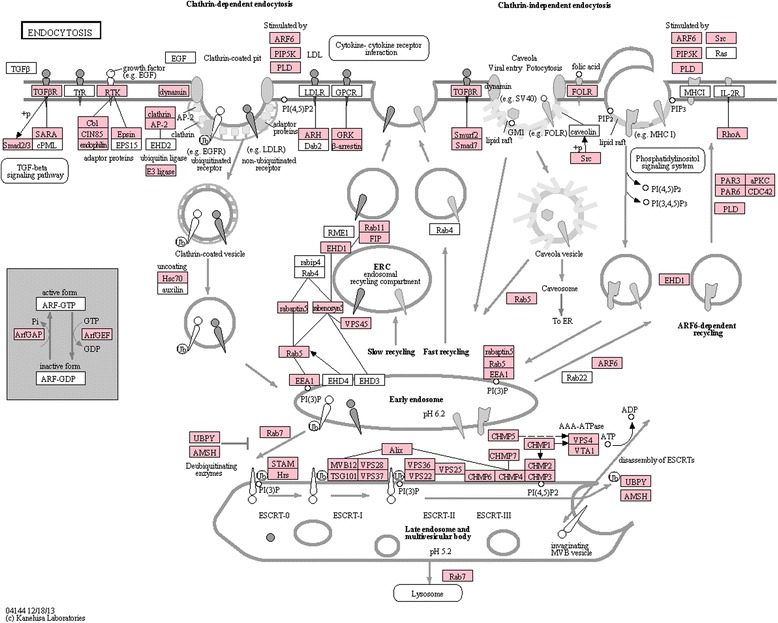
Endocytosis pathway. Overview of the KEGG endocytosis pathway. Components with homologues in the *Carcinus maenas* transcriptome are highlighted in pink

**Fig. 7:**
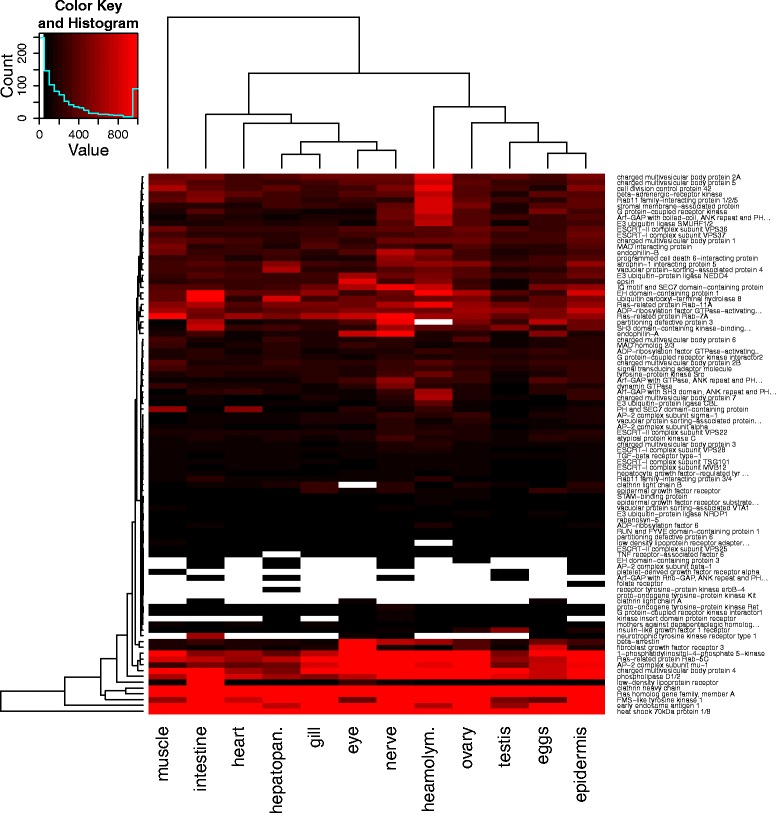
Endocytosis pathway expression. Expression of endocytosis pathway components in twelve *Carcinus maenas* tissues. The expression values are in FPKM, values of 0 are coloured white and values over 1000 FPKM are binned together

The endocytosis pathway plays a crucial role in viral challenges. Whereas some viruses are able to enter the cytosol directly, the majority require uptake via endocytosis [35]. Viral particles can enter endosomes via various endocytotic mechanisms (e.g. clathrin-mediated endocytosis, caveolar-mediated endocytosis, or micropinocytosis). Decreasing pH in the endosome environment is a cue to the viral particles, which then penetrate into the cytosol [35]. This indicates that there are important interactions between components of the endocytosis pathway and viral proteins, e.g. cellular Rab7 can interact with the VP28 protein of the White Spot Syndrome Virus [56]. Therefore, information on the sequences and expression of the *C. maenas* endocytic system may aid in the study of viral infection. The mechanisms of endocytosis, maturation of endosomes and related signalling molecules are depicted in the KEGG pathway shown in Fig. 6. The number of identified components demonstrates that *C. maenas* contains an endocytic system that closely resembles this canonical KEGG pathway. The KEGG annotation did not yield transcripts similar to caveolin, an important constituent of caveolar-mediated endocytosis. However a tBLASTn search of NCBI caveolin protein sequences in the transcriptome identified similarity between ‘comp141181_c0_seq1’ and caveolin-3-like isoform *X*2 (XP_006615923.1, *Apis dorsata*, e = 1e−15). Expression of components of the endocytosis pathway is visualized in Fig. 7, and most of these components were expressed across all tissues. The muscle tissue showed an endocytosis expression profile that differs from the other tissues.

## Conclusions

We produced an assembled transcriptome for *C. maenas* that consists of 153,699 loci and 212,427 transcripts and provides a significant molecular resource for wide studies into both basic and applied biology for this species. Comparisons run in the NCBI-nr database showed 30 % of *C. maenas* transcripts had significant homology against known sequences, but a large number were novel transcripts that have yet to be characterized. Expression analysis revealed tissues and organ transcript specificity that mapped with gene ontology annotations relating to specific tissue/organ-related functions. Of particular relevance for studies into pathogenesis and disease, we identified the presence of a series of known targets and functional pathways including the RNAi pathway, Toll-like receptor signalling, IMD and JAK-STAT pathways that form part of their innate immune system.

## Methods

### mRNA preparation

Four individual *Carcinus maenas* were collected from Newton’s Cove, Weymouth, UK and placed on ice prior to dissecting tissues and organs of interest (including gill, hepatopancreas, epidermis, eyes, intestine, haemolymph, muscle, heart, nerve, ovary, testis and eggs). All tissues and organs were immediately snap-frozen in liquid nitrogen and transported to the University of Exeter for sample preparation and analysis.

RNA was extracted using Qiagen’s miRNeasy mini kit, with on column DNase digestion, according to the manufacturer’s instructions. RNA quality was measured using an Agilent 2100 Bioanalyzer with RNA 6000 nano kit (Agilent Technologies, CA, USA). cDNA libraries for each tissue were constructed using 2.5 μg of RNA pooled from the four sampled individuals. ERCC Spike-In control mixes (Ambion via Life Technologies, Paisley, UK) were added to control for technical variation during sample preparation and sequencing, and analysed using manufacturer’s guidelines. mRNA purification was performed via poly (A) enrichment using Tru-Seq Low Throughput protocol and reagents (Illumina, CA, USA). Finally, cDNA libraries were constructed using Epicentre’s ScriptSeq v2 RNA-seq library preparation kit (Illumina). Each tissue was labelled with a unique barcode sequence to enable multiplexing of all samples across one lane whilst ensuring sequencing data from each tissue could be separated for analysis. Sequencing was performed on an Illumina HiSeq 2500 with the 2 × 100 bp paired-end read module.

### Transcriptome assembly

Prior to transcriptome assembly, the sequence reads were processed to remove those with low confidence (as assigned by the sequencer). The first 12 bp were trimmed from the reads to remove bias caused by random hexamer priming [57] and Illumina adapters were removed using Trimmomatic [58]. Trimmomatic was also used for quality trimming of the 3' end of the reads using a sliding window (4 bp with a minimal Phred quality of 30). Reads shorter that 70 bp were discarded. Only read pairs where both reads passed the desired quality threshold were retained. Read pairs of all tissue libraries were pooled and used for *de novo* transcriptome assembly using the Trinity (2013-02-25 release) software package [9]. Transcripts with a length of 200 nucleotides or less were removed from the assembly. General transcriptome statistics, including maximal transcript length, mean transcript length and N50, of the resulting transcriptome were calculated with a custom R script. This Transcriptome Shotgun Assembly project has been deposited at DDBJ/EMBL/GenBank under the accession GBXE00000000. The version described in this paper is the first version, GBXE01000000.

### Transcriptome characterization

The Trinotate suite (2013-08-26 release) [59] was used to annotate transcripts. Peptide coding regions were found through transdecoder and BLASTp v 2.2.28 (release 2013-07, e-value cutoff of 1e-5) was used to find sequence homology to UniProt/SwissProt. HMMR 3.1.b1 [60] and the Pfam database (version 27.0) were used to identify conserved protein domains. Additionally transmembrane regions were predicted with TMHMM-2.0c [61] and potential signal peptides identified with SignalP 4.1 [62]. Furthermore, homology searches were performed using BLASTx v 2.2.28 against the NCBI non-redundant (nr) protein database with an e-value cutoff of 1e-3 and BLASTn against all available *C. maenas* ESTs in the NCBI database (2013-05-10; 15,558 ESTs in total), with an e-value cutoff of 1e-3 and retaining the best 20 hits. The presence of highly conserved core eukaryotic genes was assessed using CEGMA 2.5 [29, 63]. Functional annotation analysis was conducted by assigning Molecular Function, Biological Process and Cellular Component Gene Ontology annotations to transcripts with BLAST2GO (v2.7.0) [30]. Finally, taxonomic classifications of the transcripts were determined and visualized using MEGAN 4 [64], and transcripts that did not map to the metazoan taxon were removed from the transcriptome assembly.

### Differential gene expression analysis

For each tissue, reads were mapped to the *Carcinus* transcriptome (including non-metazoan transcripts) using bowtie2 [65] and RSEM [32] to obtain overall transcript expression values. Differential transcript expression was performed by comparing each tissue to the other eleven tissues, treating the latter as biological replicates. The calculations were performed with RSEM based on the edgeR package [66] with a dispersion parameter of 0.4 which is recommended for analysis without replicates. Transcripts with an FDR < 0.01 were treated as differentially expressed. The lists of differentially expressed genes for each tissue were analysed for enrichment of Gene Ontology categories using BLAST2GO, and terms were deemed significant when FDR < 0.05.

### Pathway analysis

KEGG ontology groups were assigned to assembled transcripts through the KEGG Automatic Annotation Server (KAAS) web service [33]. Next, the presence of components of reference pathways related to immune responses, including the toll-like receptor signalling pathway (map04620), TNF signalling pathway (map04668), JAK-STAT signalling pathway (map04630) and the endocytosis pathway (map04144) were visualized through the KAAS web service [33].

Since KEGG is focused on vertebrate pathways an additional, more flexible, pathway annotation strategy was required. For identification of a pathway component (e.g. Spätzle in the invertebrate Toll signalling pathway) the following steps were followed: 1. Protein sequences for the component were downloaded from the NCBI protein database based on a search query. 2. These sequences were used as input in a tBLASTn search against the assembled transcriptome (cut-off 1e-20). 3. For every transcript with BLAST hits, a filter was applied to select the best three query sequences based on first taxonomic distance to a reference taxon (tax_id = 6759, *Carcinus maenas*) and secondly the e-value. 4. When necessary, manual filtering to remove irrelevant sequences that were returned from NCBI. An R-script that performs this analysis is supplied in Additional file 9.

Expression of pathway components was derived by adding the RSEM-derived FPKM values for transcripts that were annotated to the component (either through KEGG annotation or the annotation stratagem explained above).

### Availability of supporting data

The data set supporting the results of this article is available in the genbank Transcriptome Shotgun Assembly Sequence Database repository (http://www.ncbi.nlm.nih.gov/genbank/tsa) under the accession GBXE00000000. The version described in this paper is the first version, GBXE01000000.

## Additional files

Additional file 1:
**Cmaenas_transcript_lengths.pdf.** This file contains a histogram of transcript lengths to illustrate the presence of fragments and full length transcripts in the transcriptome. Additional file 2:
**Cmaenas_trinotate_annotation_report.txt.** Output of the Trinotate annotation pipeline, tabular format. This file contains annotation information derived from the Trinotate annotation pipeline as decribed in the Methods section. Additional file 3:
**Cmaenas_NCBIblastx.txt. Blastx results of transcripts to NCBI nr database, tabular format.** This file contains information on sequence similarity between transcripts in the transcriptome and sequences in the NCBI non-redundant database. Additional file 4:
**Cmaenas_transcriptome_GO_annot.txt. **Transcript Gene Ontology annotation, tabular format. This file contains Gene Ontology annotations for transcripts. Additional file 5:
**Tissue_GO_Enrichment.xlsx.** Enriched Gene Ontology terms for analyzed tissues. This file shows which Gene Ontology terms are enriched for tissue specific differentially expressed genes. Additional file 6:
**Pathway_components.xlsx.** This file contains sequence similarities between components of immune pathways and the transcriptome. Additional file 7:
**toll_pathway_heatmap.pdf.** Heatmap of expression values for components of the Toll-like signalling pathway. Additional file 8:
**imd_pathway_heatmap.pdf.** Heatmap of expression values for components of the IMD signalling pathway. Additional file 9:
**Pathway_annotation.R.** R script used to identify transcripts with significant sequence similarity to genes/proteins of interest.

## Abbreviations

WSSV: White spot syndrome virus
GO: Gene ontology
FDR: False discovery rate
IMD: Immune deficiency
MAPK: Mitogen activated protein kinase
PRR: Pattern recognition receptors
PAMP: Pathogen associated molecular patterns
GNBP: Gram-negative binding proteins
PGRP: Peptidoglycan recognition proteins
TLR: Toll like receptor
FPKM: Fragments per kilo bases of exons per million mapped reads
ALF: Anti-lipopolysaccharide factor
PO: Phenol oxidase
RNAi: RNA interference
