# Supplementary figures and images for: De novo assembly of the Carcinus maenas transcriptome and characterization of innate immune system pathways
Source: BMC Genomics. 2015 Jun 16;16(1):458. doi: 10.1186/s12864-015-1667-1 (PMC4469326; doi:10.1186/s12864-015-1667-1)

# Transcript Length Distribution

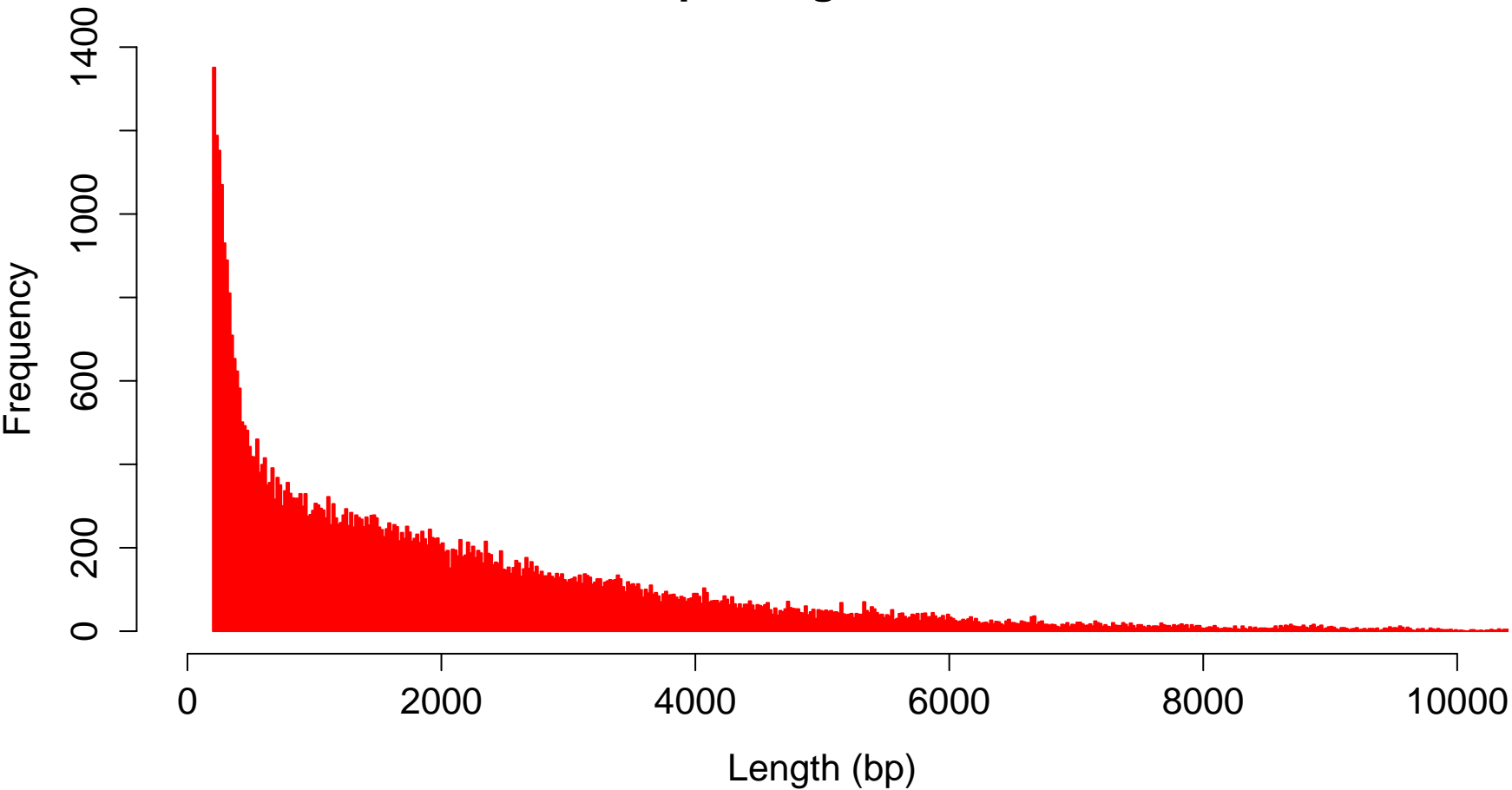

Supplement: Additional file 1: — Cmaenas_transcript_lengths.pdf. This file contains a histogram of transcript lengths to illustrate the presence of fragments and full length transcripts in the transcriptome. [file 12864_2015_1667_MOESM1_ESM.pdf]

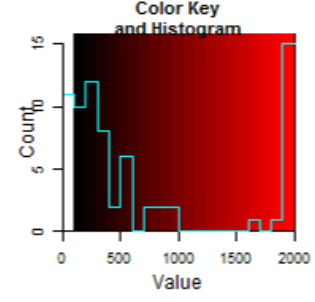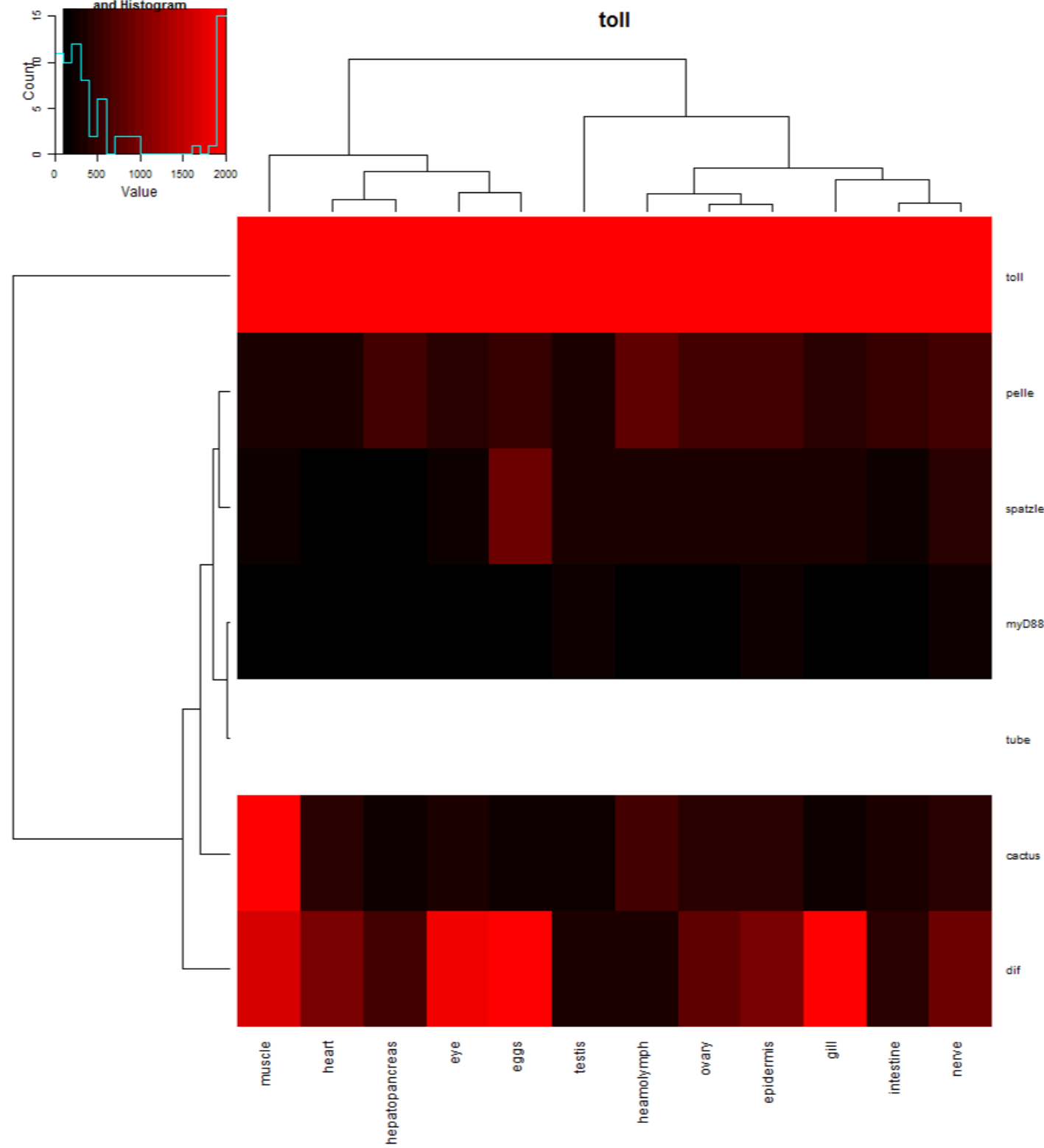

Supplement: Additional file 7: — toll_pathway_heatmap.pdf. Heatmap of expression values for components of the Toll-like signalling pathway. [file 12864_2015_1667_MOESM7_ESM.pdf]

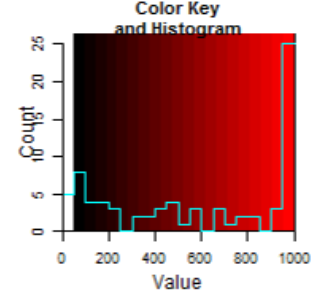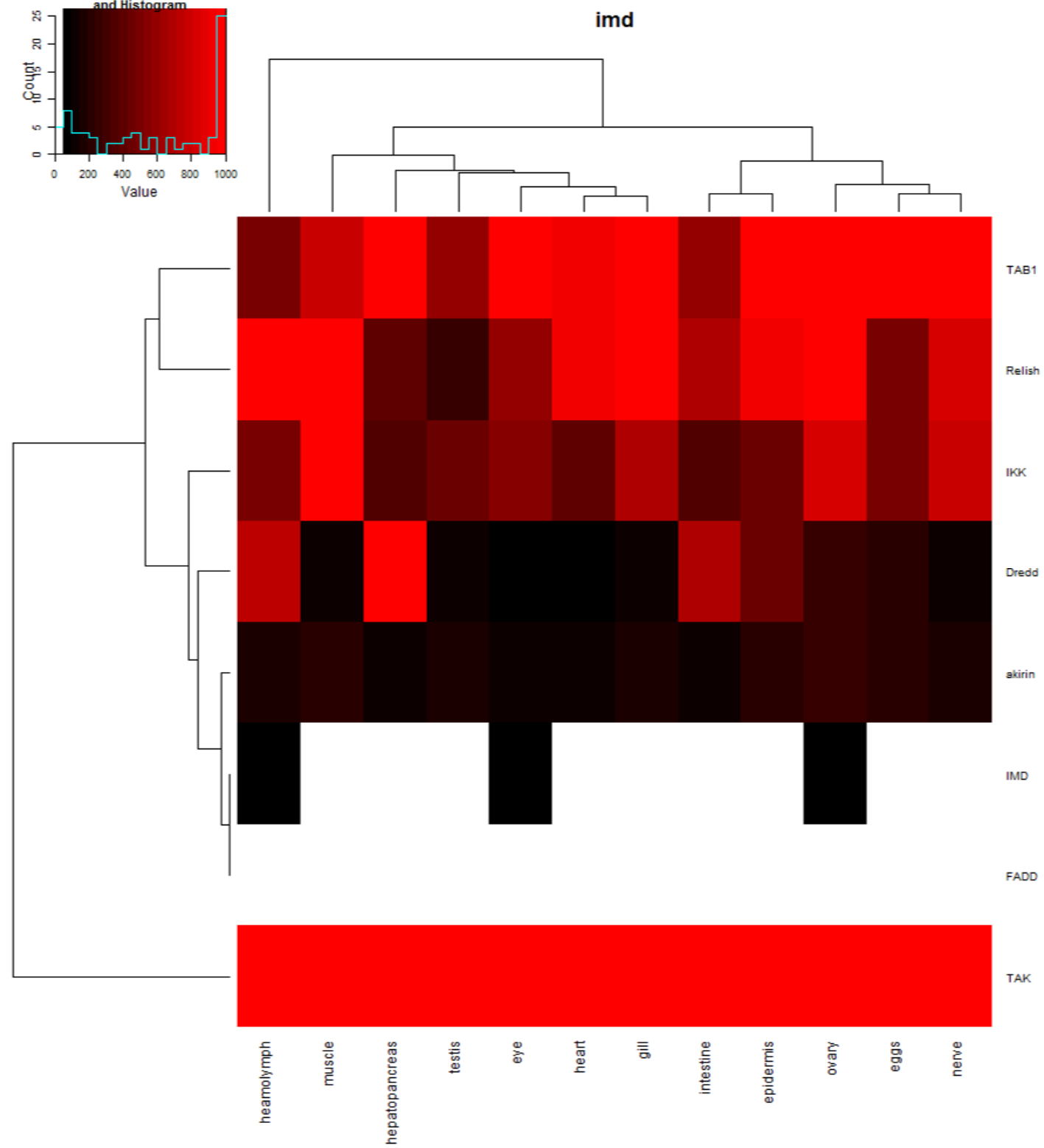

Supplement: Additional file 8: — imd_pathway_heatmap.pdf. Heatmap of expression values for components of the IMD signalling pathway. [file 12864_2015_1667_MOESM8_ESM.pdf]
